# Social frailty and brain health in nonhuman primates: Relationships between social behavior, neuroanatomy, and CSF biomarkers relevant to Alzheimer's disease

**DOI:** 10.1002/alz70856_107217

**Published:** 2026-01-08

**Authors:** Brett M. Frye, Jacob D. Negrey, Courtney L. Sutphen, Suzanne Craft, Thomas C. Register, Mark G. Baxter, Matthew J. Jorgensen, Jeongchul Kim, Richard A. Barcus, Sam N. Lockhart, Christopher T Whitlow, Carol A. Shively

**Affiliations:** ^1^ Wake Forest Alzheimer's Disease Research Center, Winston‐Salem, NC, USA; ^2^ Wake Forest School of Medicine, Winston‐Salem, NC, USA; ^3^ Emory and Henry University, Emory, VA, USA; ^4^ University of Arizona, Tucson, AZ, USA; ^5^ Wake Forest University School of Medicine, Winston‐Salem, NC, USA

## Abstract

**Background:**

In humans, age‐related accumulations of physical deficits are often characterized by frailty indices, which predict increased risk for neurodegeneration characteristic of Alzheimer's disease (AD). However, social capabilities also may diminish, with considerable variation in inter‐individual trajectories of decline and AD risk. Social frailty has recently been proposed in humans and mice to characterize the severity of social impairments with age; however, no index of social frailty exists for nonhuman primates (NHPs). This represents a significant gap in the literature, as NHPs provide important translational opportunities to study brain‐body relationships that promote healthy versus pathological aging.

**Method:**

Here, we developed a novel index of social frailty in female vervet monkeys (*Chlorocebus aethiops sabaeus*) using six components of social behavior measuring positive interactions, agonistic interactions and social isolation. Members of this study comprise the Wake Forest Aging Vervet Cohort, which includes 30+ captive females ranging from middle‐ to very old age (8‐29 years). Subjects live with their families in large, multi‐matrilineal social groups. We first tested whether social frailty in vervets recapitulated age‐related changes observed in humans. Next, we determined the cross‐sectional relationships between social frailty and neuroanatomy derived from structural MRI. Finally, we tested whether social frailty predicted biomarkers associated with AD (MesoScale Discovery Platform: Ab40, Ab42, Ab42:40, ptau181, NfL, and GFAP) in CSF at approximately one year follow‐up.

**Result:**

The social frailty index increased with age (t= 3.695, *p* <0.001). Social frailty was positively associated with CSF volumes (t= 2.163 *p* = 0.035) and negatively associated with cortical gray matter (t=‐4.261, *p* <0.001), white matter (t=‐2.591, *p* = 0.013), and cerebellar (t=‐3.716, *p* <0.001) volumes. Social frailty also predicted several CSF biomarkers associated with AD. Higer social frailty predicted higher levels of phosphorylated tau (t=2.304, *p* = 0.024) but lower Ab42 (t=‐2.345 *p* = 0.022) and Ab40 (t=‐2.138, *p* = 0.036). Social frailty was also associated with elevated NfL and GFAP in CSF; however, the associations did not reach statistical significance (*p*'s <0.10).

**Conclusion:**

Like humans, some vervets may exhibit increasing socially frailty with age. These unhealthy aging trajectories ‐ characterized by fewer positive interactions, social isolation, and agonistic interactions ‐ are associated with AD neuroimaging and biofluid biomarkers.

